# Roles of steroid receptors in the lung and COVID-19

**DOI:** 10.1042/EBC20210005

**Published:** 2021-11-26

**Authors:** Damien A. Leach, Greg N. Brooke, Charlotte L. Bevan

**Affiliations:** 1 Division of Cancer, Imperial Centre for Translational and Experimental Medicine, Imperial College London, Hammersmith Hospital Campus, London W12 0NN, U.K.; 2 School of Life Sciences, University of Essex, Wivenhoe Park, Colchester, Essex CO4 3SQ, U.K.

**Keywords:** androgen, COVID-19, estrogens, nuclear receptors, sars-cov-2, steroids

## Abstract

COVID-19 symptoms and mortality are largely due to its devastating effects in the lungs. The disease is caused by the SARS (Severe Acute Respiratory Syndrome)-CoV-2 coronavirus, which requires host cell proteins such as ACE2 (angiotensin-converting enzyme 2) and TMPRSS2 (transmembrane serine protease 2) for infection of lung epithelia. The expression and function of the steroid hormone receptor family is important in many aspects that impact on COVID-19 effects in the lung – notably lung development and function, the immune system, and expression of TMPRSS2 and ACE2. This review provides a brief summary of current knowledge on the roles of the steroid hormone receptors [androgen receptor (AR), glucocorticoid receptor (GR), progesterone receptor (PR), mineralocorticoid receptor (MR) and oestrogen receptor (ER)] in the lung, their effects on host cell proteins that facilitate SARS-CoV-2 uptake, and provides a snapshot of current clinical trials investigating the use of steroid receptor (SR) ligands to treat COVID-19.

## Introduction

The COVID-19 pandemic potentially has a degree of gender bias in terms of infection and mortality rates. As such there is growing interest in the roles of major sex hormones in lung biology, infection mechanisms, and immunological responses. Historically, the most well-studied of these three factors has been the role of hormones in immunological responses. However, in response to the COVID-19 pandemic, there has been an increase in the number of publications investigating how both male and female sex hormones control various aspects of lung biology and function, as well as their potential roles in regulating genes/proteins within host cells that are essential for viral infection.

Within this review, we will be focussing on steroid receptors (SRs), a subfamily of the nuclear receptors, due to the potential steroid-driven differences in COVID-19 pathology. Rodent studies have shown that, throughout embryonic development and sexual maturation, the lung structure and functional output can be affected by ligands of the androgen receptor (AR) and glucocorticoid receptor (GR), and to a lesser extent oestrogen receptor (ER). There is increasing evidence of gender differences in terms of COVID severity and a potential role of hormones and their target nuclear receptors in mediating COVID infections [1]. Most notably, lung cell infection by the SARS-CoV-2 virus (which causes COVID-19), as well as related viruses, has been shown to be facilitated by host cell proteins regulated by steroid hormones, especially androgens. Here we summarise the current knowledge of what different SRs do in lung epithelial biology, and how this may influence COVID-19 infection and potential therapeutic strategies.

## Architecture of the lung

**Figure 1:**
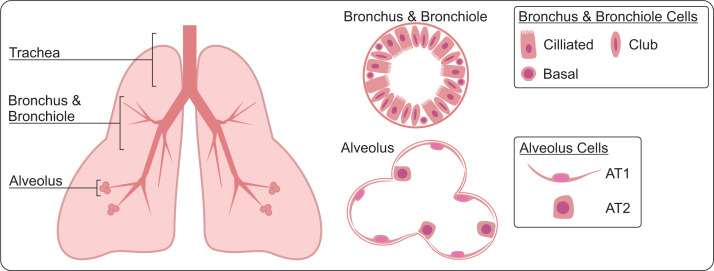
Lung architecture The lung is composed of branching tubes (bronchi and bronchioles) descending from the central trachea, leading to alveolar sacs. The tubules and alveolar sacs are composed of specialised cells which allow for gaseous exchange. AT1 - alveolar type 1, AT2 - alveolar type 2.

The lung makes up the lower respiratory tract. Emanating from a central cartilaginous tube, the trachea, tubes of bronchi span out into smaller bronchiole tubes, which culminate in alveolar sacs where oxygen/carbon dioxide exchange occurs (Figure 1). The micro-architecture of the bronchi and bronchiole tubes is composed of ciliated cells, club cells, and neuroendocrine cells lining the inside of the tubes. The trachea is similarly composed of such cells, but mucus-producing goblet cells are also found there. The alveoli are highly vascularised hollow cups, mostly composed of very thin and long squamous epithelial cells (alveolar type 1, AT1 cells) that act as an exchange surface for gases between the blood and alveolus, also cuboidal cells (alveolar type 2, AT2, cells) which secrete surfactant, a liquid that enhances gaseous exchanges.

## SRs in the lung

Although research investigating SRs in the lung is limited, the lung is a hormone responsive/targeted organ and receptors for the major steroid hormones (aforementioned AR, GR, ERs (ERα and ERβ) also progesterone receptor (PR) and mineralocorticoid receptor MR)) are reported to be expressed and functionally active – as well as a number of enzymes involved in steroid hormone metabolism. Various aspects of lung development and function have been linked to SR activity. In the developing mouse lung, the androgens testosterone and dihydrotestosterone (DHT), the major circulating oestrogen oestradiol (E_2_) and the androgen/oestrogen precursor, androstenedione, are all detectable [2]. DHT was only detected in male lung, whilst testosterone and androstenedione were higher in males than females, and E_2_ higher in female compared with male lungs.

In the lung, SR knockout/inhibition has been reported to modulate architecture and function. ERβ KO is reported to result in reduced lung function, whereas ERα KO created no phenotype [3]. In the Tfm (Testicular feminised) mouse model, complete lack of functional AR results in no change in lung weight and volume, but affects surfactant production [4]. GR KO results in a hypercellularity of the lung [5,6], whilst mineralocorticoid receptor (MR) KO is reported not to have a measurable effect [7]. Specific insights into the roles of SR activity in lung function are discussed throughout the manuscript.

Androgens are reported to be modulatory to the development of normal lung in both sexes: in both male and female mice, after addition of exogenous androgens subsequent lung structure and morphologies were altered, whilst the addition of anti-androgens resulted in opposing phenotypes [8]. In these studies, the addition of exogenous oestrogens had no effect in either sex, whilst anti-oestrogens had a minor effect upon lung morphology. GR also has a role in lung development, maturation, and differentiation of cell types, seemingly through GR expression in the mesenchyme [9]. In terms of lung function, the AR, ER, and PR have been reported to alter the gas exchange surface of alveoli [10–12] and to regulate genes involved in controlling gaseous exchange [13]. Pulmonary surfactant is a lipid complex important to airway integrity and compliance, whilst also having an innate immune function. Its production by AT2 cells is reportedly inhibited by the AR [4,14,15] and stimulated by ERα/β [16], whilst GR is reported to have both pro- and inhibitory effects on production of surfactant components [15,17,18]. This effect on surfactant production is distinct from the effects that AR and ER have on development, where both promote maturation and maintenance/turnover of alveolar sacs and cells [14,19,20]. Taken together, it is apparent that the lung is a steroid hormone-regulated organ, with lung architecture, morphology, and function modulated by a number of sex hormones as well as glucocorticoids.

## COVID-19 pathogenesis in the lung

**Figure 2:**
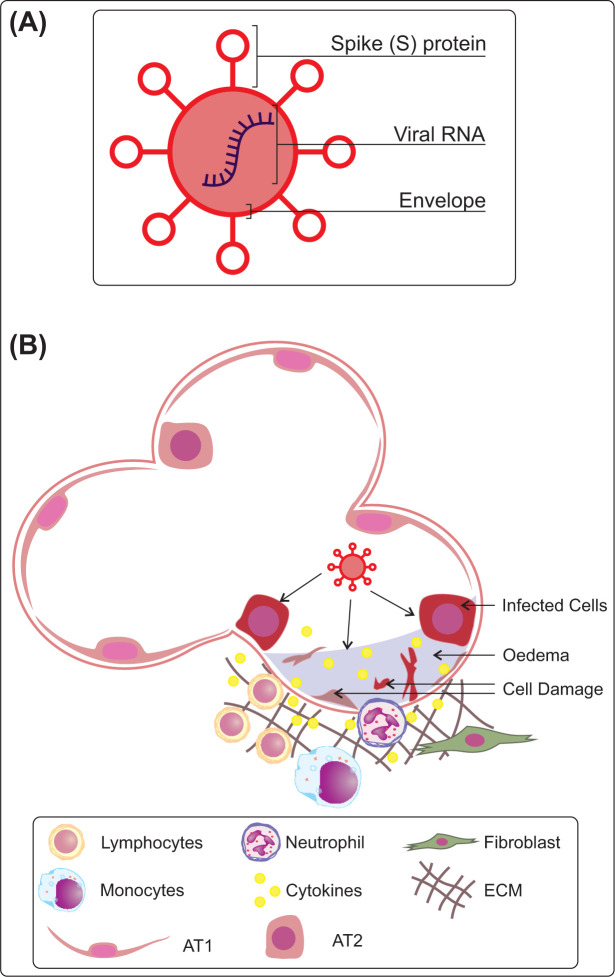
SARS-CoV-2 lung entry (**A**) Representative image of basic COVID-19 structure. (**B**) Lung damage caused by COVID. COVID infects epithelial cells in the alveolar sac, increasing viral load within infected cell, leads to cell damage/death causing immune activation, cytokine release, fibrotic responses.

SARS (Severe Acute Respiratory Syndrome)-CoV-2, the virus responsible for COVID-19, is a sense RNA virus, encapsulated in a viral envelope which is laden with a crown of spike glycoproteins (S-proteins) [21,22] (Figure 2A). These spike proteins are essential for viral entry [22]. Within the spike protein are two functional substrates which allow interactions with the host cell membrane proteins and fusion between the viral and cellular membrane. To achieve viral entry, two proteins are required on the cellular surface of the host cells, ACE2 (angiotensin-converting enzyme 2) and TMPRSS2 (transmembrane serine protease 2) [23,24]. Both proteins are expressed in many organs throughout the human body, but within the lung, expression is highest in type 1 and 2 alveolar epithelial cells (AT1 and AT2) [25–27]. These cell types are reported as the main cells targeted by COVID-19 (Figure 2B).

The viral S-protein binds to the ACE2 protein as its primary host cell receptor [21,28–30]. To allow this to happen, first TMPRSS2 cleaves both the spike and ACE2 proteins, which allows for the fusion of the viral and cellular membrane, culminating in membrane engulfment and viral entry into the host cell. Within the now infected host cell, the virus reproduces and is subsequently released from the host cell to infect surrounding cells. Demonstrating its importance in this process, TMPRSS2 expression is associated with coronavirus infection severity [31] and TMPRSS2 inhibition prevents SARS2 lung cell entry [32].

Severe lung symptoms of COVID-19 include development of alveolar injury, oedema, fibrotic responses, acute respiratory distress syndrome (ARDS), decreased blood oxygen saturation, progressive pneumonia and eventual organ failure (Figure 2B) [33,34]. During pathological progression an overactivity of the immune system in response to SARS-CoV-2 infection is characterised as a means by which some of these severe symptoms occur [34,35]. The virus is displayed to antigen-presenting cells from infected epithelial cells, macrophages or dendritic cells, resulting in activation of lymphocytes and the mass secretion of cytokines, which attracts accumulation of immune cells. This increase in cytokines, referred to in the most severe cases as a cytokine storm, includes IFN-α, IFN-γ, IL-1β, IL-6, IL-12, IL-18, IL-33, TNF-α, TGFβ, CCL2, CCL3, CCL5, CXCL8, CXCL9, CXCL10 etc [33,35,36]. The cytokine storm causes the immune system to attack the lung and cause injury/ARDS [37]; single cell analyses of bronchoalveolar lavage suggest neutrophils and macrophages are involved in this tissue destruction [38]. The resulting damage from these occurrences impairs breathing and can ultimately result in patient death.

Increasingly, it is apparent that whilst lung is the tissue most devastated by COVID-19, other organs are also targeted including the skin, cardiovascular system, kidneys, and male genital tract. As our treatments of lung injuries caused by COVID-19 improve, the effect on other organs is likely to be a growing concern, thus it will be vital to understand which other organs are potentially targeted by and susceptible to the virus. For example, the prostate and male reproduction tract are known to express high levels of TMPRSS2. COVID-19 can infect these cells [39], and is reportedly detectable in the semen of patients [40], but the long-term effect of this is yet unknown.

## Gender and COVID-19

The reported gender disparities in severity of COVID infections suggests the possibility of sex hormones and associated SRs having a role in how the host is affected by and/or reacts to SARS-CoV-2. Globally, overall infection rates may be higher in males than females, and there is also a difference based on menstrual status, with post-menopausal women having worse outcomes than matched pre-menopausal [41–43]. However, the gender effect is controversial as there are also data which suggest that infection rates are similar between genders [44]. What is apparent though, is that COVID-19 severity and morbidity is significantly worse in males [42,44–46]. Analysis of 28854 patients in Brazil found associations between male gender and increased COVID mortality rates [47]. In a separate analysis by He et al. [48] of 33 separate studies, 27 indicate higher COVID infection rates in males, whilst another independent analysis of 34 studies, combining 5057 patients, showed mortality was significantly higher in males (odds ratio (OR) = 3.4, [49]). Meta-analysis of ten literature studies, which characterised symptoms, infection rates, and outcomes, also indicated an association between male gender and higher rates of infection and mortality [50]. Interestingly, in a meta-analysis of articles published in the first four months of 2020, investigations into the 85 articles that met criteria found no significant difference in infection rate between males and females, but a significant association of male gender with ‘severe’ COVID-19 (OR = 1.46), lower chances of recovery (OR = 0.72), and mortality (OR = 1.81) [51]. Another, separate, meta-analysis of 12 studies involving 281461 patients from 11 different countries indicated that males were significantly associated with mortality (coefficient = 5.1) [50]. In summary, although infection rate disparities are not yet clear, there is now a large number of studies and analyses indicating that COVID-19 severity and outcomes are significantly worse in males.

Gender-related differences in molecules involved in viral infection may contribute to these gender disparities. ACE2 expression correlates with age, and has been reported as increased in males compared with females both generally and within the lung [26,52–55]. Whether TMPRSS2 expression is higher in male lungs than female is controversial, with some studies reporting a slight but significant difference [56,57] but others reporting no significant difference [58,59]. More generally, there are also gender disparities in response to viral infections, with females producing stronger immune responses and better clearance of viral loads [60]. Male-pattern baldness (androgenic alopecia), which is linked to increased androgen activity, has been associated with increased risk of COVID-19 infections in both males and females [61–65] indicating that hormones may be involved. The potential role for hormones in COVID-19 was also shown in an analysis of females using the COVID Symptom Tracker Application in the United Kingdom: this showed COVID-19 infections and hospitalisation were significantly lower in women aged 18–45 taking oral contraceptives compared with matched women not taking oral contraceptives [66]. Of course, there are gender-associated behaviours that may affect COVID-19 infections and mortalities, for instance males are more likely to be smokers which increases ACE2 expression [1]. Overall, there does appear to be a gender and hormone bias in the severity of COVID-19 infections.

## Immunomodulation by SRs

Cells of the immune system express SRs, and hormones are known to be able to modify the transcriptional/epigenetic profile of immune cells and modulate immune responses [67–70]. Whilst this may be an important aspect in patient response to SARS-CoV-2 infection, our discussion of this aspect will be brief, as others have reviewed immune modulation by hormones in respect to COVID-19 [71].

While oestrogens and progesterone have pro-inflammatory effects, testosterone is reputed to have immunosuppressive effects, and glucocorticoids are routinely used clinically to suppress immune response through suppression of cytokines [72]. Oestrogens and progestogens promote cytokine release, potentially leading to a more responsive immune system [73]. Further, a number of steroid hormones are able to affect cytokine expression in both immune cells and non-immune cells such as epithelia. This is important as cytokines mediate immune responses in both autocrine and paracrine manners [74–76]. A further note is the gender differences in the antibody response to influenza vaccinations, and a correlation between antibody response and serum testosterone in males [77], which again points to differences in the immune responses between genders.

These brief descriptions relay that sex steroids and other steroid hormones are immunomodulatory, which may be relevant when dealing with severe COVID-19 reactions where an active immune system leads to lung damage. However, in this review, we will focus on lung cell-intrinsic mechanisms by which steroid hormone receptors may affect COVID pathology.

## Direct effects of SRs in lung cells and their role in COVID infections

**Figure 3:**
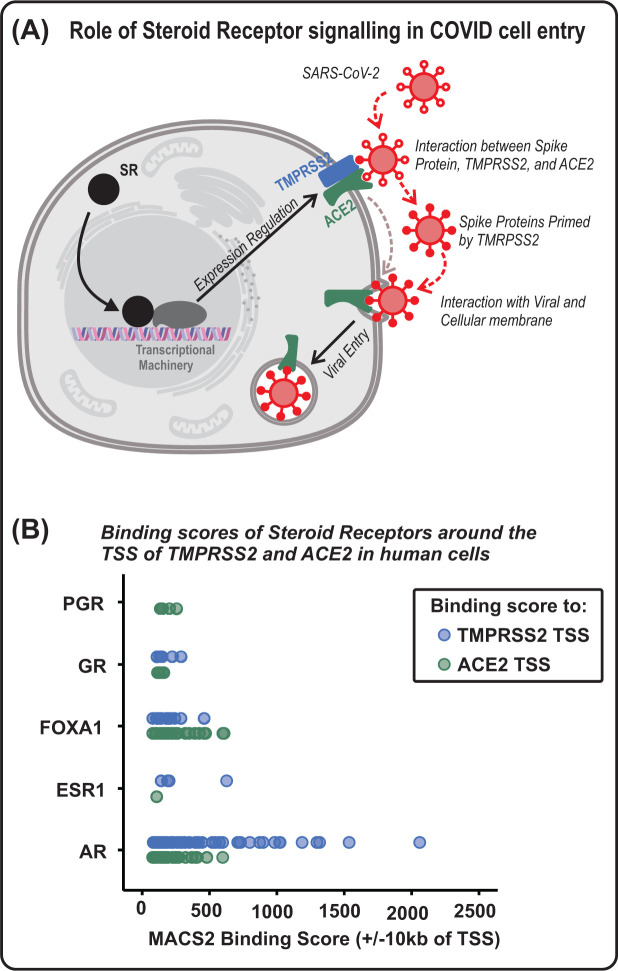
Steroid receptor regulation of host genes affecting COVID infection (**A**) Role of SRs in COVID infection of cells. SRs regulate ACE2 and TMPRSS2 expression, allowing for SARS-CoV-2 interaction with ACE2, activation of S-proteins by TMPRSS2, and viral entry. (**B**) Data from www.signalingpathways.org indicating binding scores (x-axis) of the androgen (AR), glucocorticoid (GR), progesterone (PGR), and estrogen (ESR1), receptors within an area of 10 kb up- or downstream from the transcription start site (TSS) of the ACE2 (green) and TMPRSS2 (blue) genes in multiple human cell types. Binding scores for the pioneer factor, FOXA1, are also provided.

Within this section, we discuss the mechanisms by which SRs regulate proteins required for viral entry (Figure 3A). It is important to note that the regulatory regions of genes involved in COVID viral entry (i.e. TMPRSS2 and ACE2) have response elements for AR, ER and GR that allow for SR binding, as depicted in Figure 3B, identified using publicly available data (www.signalingpathways.org [78,79]).

### AR

Given the potential male bias in COVID-19 mortality, androgens are being keenly assessed for involvement in SARS-CoV-2 infection and in the lung. Androgens, acting through AR, have key roles in lung development in both genders [8]. In other diseases of the lung, such as lung cancer, higher testosterone or DHT is associated with increased incidence of lung cancer in older males [80]. Androgens are also reported as having a role in ARDS, which is more prevalent in males compared with females, and a role for mediating fibrotic and immune responses in the lung, such as collagen deposition and cytokine levels [81,82].

Given that men with higher levels of circulating androgens appear to have greater risk of severe COVID-19 symptoms [61–64], a converse cohort of patients to be investigated further are men who receive the weak adrenal androgen dehydroepiandrosterone (DHEA) as a means of counteracting low serum testosterone levels, to determine whether this affects COVID-19 infections and severity [83]. Initial population studies, undertaken when the disease first swept through Italy, indicated that in prostate cancer patients, those patients taking anti-androgen therapy had lower infection rates and lower risk of hospitalisation [84]. A similar study conducted in the United States with 58 prostate cancer patients found significant inverse association between anti-androgen therapy and hospitalisation and the need for respiratory assistance with ventilators [85]. Whilst there may be other contributing factors that could influence this finding (such as increased shielding of these patients), the evidence from these different conditions/diseases plus the reported gender disparities support the potential benefit of reducing androgen action – indeed of targeting the AR – in COVID-19 patients.

A number of studies have now investigated mechanisms by which androgens may influence COVID-19 outcomes. Regulation of ACE2 expression is one means by which androgens have been proposed to regulate viral entry. Markers of ACE2 activity, plasma renin activity and angiotensinogen expression, are increased by androgens [86]. In prostate cancer cell lines, androgens and anti-androgens have been reported to regulate ACE2 expression [39,87], and studies in mouse whole lung tissue support this [88]. However, in other studies, ACE2 was not seen to be androgen- or antiandrogen-regulated in lung cell lines or mouse lung tissue at the RNA level [13,56]. Another means of androgen/AR signalling influencing viral entry is by TMPRSS2 regulation. TMPRSS2 is a well-known transcriptional target of AR in prostate cancer cells, and has recently been shown to be regulated by androgens and anti-androgens also in lung cells and mouse lung tissue [13,56]. Anti-androgens can also inhibit both pseudotyped and live SARS-CoV-2 viral entry in prostate cancer cells [39] and, more importantly, human lung cells [56]. Furthermore, anti-androgen inhibition of SARS-CoV-2 entry into lung cells was shown to be concomitant with down-regulation of TMPRSS2, and to occur even in cells stably overexpressing ACE2, supporting a pivotal role for TMPRSS2 down-regulation in the inhibitory effect of anti-androgens on virus entry. The specificity of this inhibitory effect of anti-androgens is emphasised by the presence of androgen response elements (AREs), known to bind AR, within the regulatory regions of TMPRSS2 (Figure 3B) [56,89].

Another potential means by which androgen may affect COVID-19 outcomes is through surfactant production. As alluded to earlier, androgens regulate alveolar cell production of surfactant, which coats the alveolar sac and has an immunologic role. Two of the protein components of surfactant, SP-A and SP-D, interact with pathogens promoting uptake by immune cells [90]. Disruption of surfactant has been reported in ARDS and in COVID-19 patients [91], and rectifying this disruption through exogenous surfactant has been hypothesised as a means of treating COVID-19 patients to minimise severity [91,92]. Further studies are required to see if anti-androgen therapy associates with or enhances surfactant levels.

The most direct way to inhibit androgen action is direct inhibition of the AR. A number of anti-androgens, which directly bind to the ligand-binding domain of AR and prevent its activation by ligand [93], are used to treat prostate cancer as well as other diseases such as polycystic ovarian syndrome, alopecia and are being trialled for breast cancer [94]. There are currently several clinical trials actively assessing the effectiveness of targeting AR in treating COVID-19 patients using new or re-purposed anti-androgens. Repurposed drugs being trialled are anti-androgens enzalutamide (NCT04475601), bicalutamide in combination with the TMPRSS2 inhibitor camostat (NCT04652765), and the anti-androgen/anti-hypertensive spironolactone (NCT04345887) to assess if blocking AR activity for several days in COVID-19 patients improves outcomes. In another trial (NCT04446429), 262 COVID-19 positive male participants in Brazil were tested for the effectiveness of the new anti-androgen proxalutamide in preventing hospitalisation within 30 days. In preliminary reporting from this trial, no patients receiving proxalutamide underwent hospitalisation, whilst in the control arm 27.3% of patients were hospitalised. A larger follow-on trial has been listed, of both men and women (NCT04446429). We await with interest the peer-reviewed publication of results from this and the other trials as to whether targeting AR action will be of clinical benefit.

There are also clinical trials evaluating inhibition of IL-6 (using Tocilizumab, an IL-6 inhibitor, and Sarilumab, an IL-6 antagonist) [95]. These are of particular interest here as IL-6, in addition to being a powerful cytokine, is known to regulate AR activity [96]. Dampening of lung fibrosis is also being investigated as a means of aiding COVID-19 recovery/minimising COVID-19-induced damage [97]; as AR is expressed in lung fibroblasts [56], and as we know from the prostate, AR expressed in fibroblasts modifies fibrotic activity [98], so this too may be a means by which anti-androgens could benefit COVID-19 patients.

### ER

Similar to the AR, there are two pathways by which ER activity may influence COVID-19 infections: regulation of host cell proteins and/or regulation of the immune system. In mouse studies of SARS‐CoV (the highly related coronavirus responsible for the 2003 SARS pandemic), ovariectomised or antioestrogen-treated female mice had more severe infections than control mice [99], which supports a potential protective role of oestrogens, also suggested by the reduced COVID-19 in women taking oral contraceptives previously alluded to [66]. In support of regulation of host cell proteins being involved in this, cardiovascular ACE2 activity is higher in female mice [100] and oestrogens regulate ACE2 expression in a range of tissue types [101–104]. In non-lung cells, such as prostate, breast and kidney, oestrogen may also inhibit TMPRSS2 expression [105–107]. In primary normal human bronchial epithelia, ER has been reported to inhibit expression of ACE2, but not TMPRSS2 [102], whilst in human-derived A549 lung cells, E_2_ could inhibit TMPRSS2 expression [107]. In animal models, oestrogens and ER modulators have been reported to reduce ACE2 expression and activity [108,109]. Further, ER activation in lung stromal smooth muscle cells down-regulated ACE2 in male and females [110], the relevance of this remains unclear, but it may be involved in fibrotic responses such as are seen in advanced COVID-19. Interestingly, in kidney Vero cells, 17β-E_2_ reduced SARS-CoV-2 viral load within cells, which was associated with decreased TMPRSS2 levels but not ACE2 [106].

In terms of ER ligands, on the basis that agonists inhibit TMPRSS2 expression it would be ER agonists rather than, as is the case for AR, antagonists that may be useful in COVID-19. However, the effect of ER agonists on ACE2 expression and downstream effects must also be clarified. Currently, there are no reported results from clinical trials looking into oestrogens to treat/prevent COVID-19 infections, but several are recruiting for use of oestrogen patches and oestrogen modulation therapies (NCT04359329, NCT04801836, NCT04389580).

### PR

Progesterone activity is directly influenced by ER activity (PR is an ERα target gene), so the direct role of PR in the lung and COVID-19 may be difficult to unravel. PR is expressed in multiple cell types throughout the lung [111,112]. In the mesenchyme of the lung, progesterone is able to affect vasodilation of the blood vessels, and controls contraction/relaxation of smooth muscle cells to aid inhalation [113]. These roles of progesterone may be exploitable as a means of reducing COVID-19 symptoms. In lung epithelial cells, PR regulates the expression of a number of inflammatory cytokines as well as regulating immune cell activation [76,113–115]. In uterine tissue, progesterone is able to reduce ACE2 expression, more so than oestrogen treatment [116]. No current publications offer a specific role for PR in regulating ACE2 or TMPRSS2 in lung epithelia, although as PR binds to the same response elements as AR and GR, direct regulation is possible. Indeed in exogenous models, progesterone does elicit a strong signal in TMPRSS2-based reporter systems [117]. Despite this, the above-stated roles of PR in directly and indirectly regulating immune activity may provide benefit to patients; as such a small (*n*=40) trial (NCT04365127) has been completed for testing the effects of progesterone on men hospitalised with COVID-19, though at this time results have not been published.

### GR

A number of dehydrogenases and other genes involved in glucocorticoid synthesis are expressed in the developing lung [118]. The function of GR in the lung appears to inhibit cell proliferation, potentially in both epithelia and mesenchyme. KO of GR in developing lung is associated with hypercellularity in alveolar sacs, leading to airway collapse [5,6]. More specifically, KO in the epithelia leads to either mild hyperplasia or no effect in male and female mice, but KO in the mesenchyme consistently leads to severe hyperplasia within airways [9]. It is also suggested that KO of GR inhibits the differentiation of the lung mesenchyme, which in turn prevents development of AT1/AT2 alveolar cells [119]. KO of HSD11B1 (11β-hydroxysteroid dehydrogenase), a metabolising enzyme involved in the gonadotropin axis, reduces surfactant synthesis from AT2 cells [120–122]. Exogenous glucocorticoids stimulate expansion of airways' lumen diameter, reduce the amount of mesenchyme present, and decrease fibrotic response and ECM deposition [9].

GR expression and function is already well noted in other lung diseases. GR expression is significantly increased in the lungs of patients with chronic obstructive pulmonary disease (COPD), but decreased in interstitial lung disease (ILD) [123]. In ARDS and lung injury, synthetic glucocorticoids improve lung physiology, with reductions in inflammation and injury induced by macrophages [124].

The widespread use and effectiveness of GR ligands in many lung diseases has led to trials in COVID-19 patients with severe symptoms. Dexamethasone reduced mortality of COVID-19 patients receiving respiratory support [125–127], with no advantageous effect noted in patients not requiring such support. Dexamethasone was also able to reduce the time that patients required ventilators and increased recovery rates [128]. Dexamethasone is now a common worldwide treatment for patients hospitalised with COVID-19. Its main source of action appears to be inhibition of cytokine production, dampening the cytokine storm. Such treatment appears to be effective in reducing viral loads, and enhancing patient recovery, although there are potential side effects of the concomitant immunosuppression [129,130]. Further trials are underway to test the effectiveness of other, inhalable (thus routed directly to the lung and potentially reducing side effects), glucocorticoids, such as Budesonide (NCT04416399, NCT04355637). The effect of inhaled glucocorticoids may involve the immunomodulatory effects of these compounds [131]. There may be an aspect of GR signalling affecting viral entry into lung cells as well. Dexamethasone has been reported in mouse placental studies to inhibit ACE2 expression [132,133]. In mouse and human COPD models, inhaled corticosteroids were reported to inhibit ACE expression but no change in TMPRSS2 expression was associated with their use [134]. In asthma patients, inhaled corticosteroids are also able to reduce ACE2 expression [135]. Again, GR can bind to the same response elements as AR and PR, and we and others have shown there is detectable binding of GR in the TMPRSS2 regulatory region [56,136], and dexamethasone does inhibit TMRPSS2 expression in the A549 lung cell line [13]. It is possible that GR activity, aside from modulating the immune system, may also influence ACE2 and TMPRSS2 expression in lung cells with potential to affect SARS-CoV-2 uptake/infection. As there is documented cross-talk between GR and AR, there is also the possibility of dexamethasone inhibition of AR expression/function and testosterone production [137–139]. How this AR–GR cross-talk translates to lung tissue and COVID infection is not currently known, though emerging preclinical data suggest that simultaneously targeting both receptors may be of benefit [140].

### MR

MR is expressed in both foetal and adult lung, where it is expressed in alveolar, macrophage and endothelial cells [141–143]. In MR knockout mice, it has been reported there was no phenotypic change in the lung [7]. The normal physiological role for MR in the lung is in liquid homoeostasis, regulating ion channel expression to modulate fluid re-absorption. MR is also regulated by the ACE and angiotensin pathways [144]. In COVID-19 infected cells, MR activity is reported to be increased [144,145], where it is suggested to play an indirect role in the persistent cough associated with the disease [145]. MR antagonists are reported to inhibit pro-inflammatory genes, and increase *ACE2* mRNA expression and ACE2 activity [146–148], despite this there was no MR binding detected around ACE2, nor TMPRSS2, genes (www.signalingpathways.org [78,79]). MR and GR activity in the lung are linked, with MR activity changing with available glucocorticoid and under pressure from oxidative stress, as seen in COVID patients [118,145]. There is controversy as to the use of anti-MR agents for COVID-19 patients, with further research needed to uncouple its exact role, and whether there are specific stages within the disease when targeting MR can be of use. Spironolactone, which has anti-MR activity, is currently being used in two clinical trials (NCT04826822, NCT04643691), but results are yet to be reported.

## Conclusions

Severity of COVID-19 appears to be biased between genders with males more severely affected. This circumstantial evidence for involvement of sex steroid signalling is supported by molecular studies demonstrating effects of steroid hormones and their receptors on lung development/function and SARS-CoV-2 infection. Together, this supports use of SR ligands in COVID-19. There are several clinical trials showing an ability of steroid hormones to affect COVID-19 severity and patient recovery. This is likely due to a combination of immunomodulation and ability of SRs to mediate the expression of proteins required for SARS-CoV-2 viral infection.

## Summary

- All SRs are expressed in the lung.
- Steroid hormones are involved in lung development and maturation, with specific roles in regulates lung surfactant and gaseous exchange.
- SRs mediate immune response in the lung.
- Entry into lung cells of the SARS-CoV-2 virus, responsible for COVID-19, is facilitated by host proteins TMPRSS2 and ACE-2, expressed on the surface of lung cells.
- AR-, ER-, and GR-binding sites are present adjacent to ACE2 and TMPRSS2 genes.
- Manipulation of SR activity may play a key role in mediating SARS-CoV-2 infections and ligands such as anti-androgens have therapeutic potential for the disease.

## Open Access

Open access for this article was enabled by the participation of Imperial College London in an all-inclusive *Read & Publish* pilot with Portland Press and the Biochemical Society under a transformative agreement with JISC.

## Abbreviations

ACE2: angiotensin-converting enzyme 2
AR: androgen receptor
ARDS: acute respiratory distress syndrome
AT1: alveolar type 1
AT2: alveolar type 2
COPD: chronic obstructive pulmonary disease
DHT: dihydrotestosterone
ECM: extracellular matrix
ER: oestrogen receptor
E_2_: oestradiol
GR: glucocorticoid receptor
KO: knockout
MR: mineralocorticoid receptor
OR: odds ratio
PR: progesterone receptor
SARS: severe acute respiratory syndrome
SR: steroid receptor
S-protein: spike glycoprotein
Tfm: Testicular feminised
TMPRSS2: transmembrane serine protease 2
